# A Review on Porous Polymeric Membrane Preparation. Part II: Production Techniques with Polyethylene, Polydimethylsiloxane, Polypropylene, Polyimide, and Polytetrafluoroethylene

**DOI:** 10.3390/polym11081310

**Published:** 2019-08-05

**Authors:** XueMei Tan, Denis Rodrigue

**Affiliations:** 1 College of Environment and Resources, Chongqing Technology and Business University, No.19, Xuefu Ave, Nan’an District, Chongqing 400067, China; 2 Department of Chemical Engineering, Laval University, 1065 Avenue de la Médecine, Quebec, QC G1V 0A6, Canada

**Keywords:** porous polymeric membranes, polyethylene, polydimethylsiloxane, polypropylene, polyimide, polytetrafluoroethylene, processing, morphology, separation performance

## Abstract

The development of porous polymeric membranes is an important area of application in separation technology. This article summarizes the development of porous polymers from the perspectives of materials and methods for membrane production. Polymers such as polyethylene, polydimethylsiloxane, polypropylene, polyimide, and polytetrafluoroethylene are reviewed due to their outstanding thermal stability, chemical resistance, mechanical strength, and low cost. Six different methods for membrane fabrication are critically reviewed, including thermally induced phase separation, melt-spinning and cold-stretching, phase separation micromolding, imprinting/soft molding, manual punching, and three-dimensional printing. Each method is described in details related to the strategy used to produce the porous polymeric membranes with a specific morphology and separation performances. The key factors associated with each method are presented, including solvent/non-solvent system type and composition, polymer solution composition and concentration, processing parameters, and ambient conditions. Current challenges are also described, leading to future development and innovation to improve these membranes in terms of materials, fabrication equipment, and possible modifications.

## 1. Introduction

In the field of membrane technology, a successful membrane is a barrier to selectively transport substances of interest. Since the core concept of an excellent membrane performance lies in its final morphology, the selection of materials and fabrication techniques have a significant effect on membrane morphology. Thus, the optimization of membrane performances must go through precise control of the interactions between morphology, materials, and fabrication technology [1,2,3].

With respect to materials, porous polymeric membranes have been widely applied for industrial processes due to a combination of specific properties such as permeability, selectivity, fouling resistance, chemical and thermal stability, low cost, and easy manufacturing. To date, the most popular membranes for separation processes include polyethylene (PE), polysulfone (PSU), poly (vinylidene fluoride) (PVDF), polydimethylsiloxane (PDMS), polypropylene (PP), polyimide (PI), and polytetrafluoroethylene (PTFE) membranes. For specific materials, the membrane morphology can be tailored by the precise control of the fabrication methods and processing conditions.

For their production, porous polymeric membranes can be made by several methods. Usual approaches include phase inversion, melt-spinning and cold-stretching (MSCS), electro-spinning, track etching, and sintering, while novel technologies include phase separation micromolding (PSµM), imprinting/soft molding, manual punching, and three-dimensional (3D) printing. Each method has its own advantages, limitations, and different pore formation mechanisms to produce porous polymeric membranes. Table 1 provides a comprehensive overview of the techniques used for membrane fabrication.

Over the past half century, significant knowledge has been generated regarding membrane technology. However, to the best of our knowledge, the majority of the literature focused on a specific preparation method or a given material. As a result, understanding the relationships between the membrane morphology and the fabrication parameters is very difficult. Thus, the main objective of this review paper is to summarize the existing and the potential principles to select appropriate methods and membrane fabrication systems to produce membranes with a desired morphology, performance, and stability, as well as to select the best method(s) to obtain these properties in an efficient way for future research. In the first part of this work, PSU and PVDF membranes produced by non-solvent induced phase separation (NIPS), vapor-induced phase separation (VIPS), electrospinning, track etching, and sintering [10]. In this second part, other methods for porous membrane fabrication are presented, and a focus on two other polymers is made: PE and PDMS. Nevertheless, polymers such as PP, PI, and PTFE are also introduced.

The main contribution of this article is to emphasize the methods and the processing conditions to achieve desired morphology, performance, and stability. The paper is organized as follows. Section 2 describes the membrane characterizations, including morphological and separation performances. Section 3 presents PE membranes, while Section 4 describes PDMS membranes. Section 5 presents an overview on PP, PI, and PTFE membranes. Each polymer is described in detail with respect to its key manufacturing parameters, providing the relationships between processing (methods and conditions) and morphology control. Finally, Section 6 presents some concluding remarks and insights for future studies.

## 2. Morphological and Performance Characterization of Membranes

### 2.1. Morphological Characterization

In general, the most important morphological parameters for a membrane are porosity, pore size, pore size distribution, tortuosity, surface roughness, molecular weight cutoff, and thickness. For example, the pore size plays a critical role in the membrane classification [40,41,42]: microfiltration (50–500 nm) [42,43], ultra-filtration (2–50 nm) [42,43], nano-filtration (≤2 nm) [42,43], reverse osmosis (0.3–0.6 nm) [42,44], and forward osmosis (0.3–0.6 nm) [42,44]. Furthermore, the membrane performances directly depend on its morphology (pore size and distribution), thus morphology control is the key factor in membrane fabrication.

### 2.2. Performance Characterization

Generally, the membrane performances can be evaluated by their productivity (rate) and separation ability (selectivity). Parameters such as flux (J), permeability (P), and permeance (P’) play essential roles in the evaluation of the membrane productivity, while efficiency (separation performance) is determined by the selectivity (α) and the separation (β) factors [45,46]. However, when polarization effects are present, the observed retention coefficient (R_obs_) and the true retention coefficient (R) must be measured [47,48]. More details on membrane performance and characterization can be found in the first part of this work [10].

## 3. PE Membranes

Microporous PE membranes have stable chemical properties, good mechanical strength, appropriate permeability, rejection properties, and low cost. As one of the most commonly used microporous membranes, PE is widely used in sterile filtration for food processing, pre-filters for the production of drinking water, advanced sewage treatment, and separators for rechargeable batteries [49,50,51,52,53,54].

### 3.1. Properties of PE Membranes

**Figure 1:**
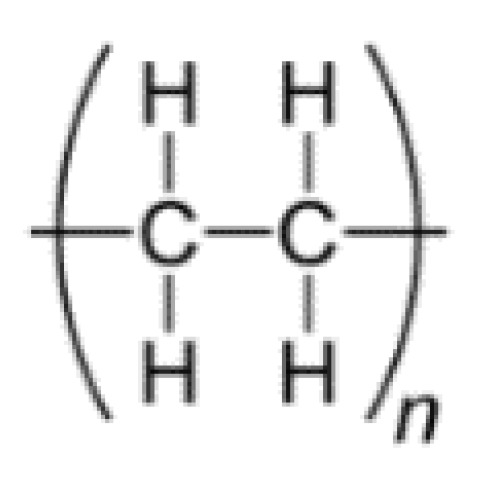
The structure of polyethylene (PE) (CAS number 9002-88-4).

In general, PE is composed of a –C_2_H_4_–repeating unit (Figure 1). The polymer is an odorless and non-toxic semi-crystalline polymer with good acid and alkali corrosion resistance, low water absorption, and excellent electrical insulation. However, PE has a relatively low melting point (around 120 °C) and low temperature resistance [continuous service temperature (CST)] of 60–80 °C for low density (LDPE) and 80–100 °C for high density (HDPE). Based on these properties, the two main methods to prepare PE microporous membranes are thermally induced phase separation (TIPS) and MSCS [20].

### 3.2. TIPS

As the main method to produce microporous PE membranes, TIPS is distinguished from other methods because the membrane microstructure can be more easily controlled [52,55,56,57,58,59,60,61,62]. For example, compared to NIPS, the homogeneous solution from which the membrane is formed is converted into a two-phase mixture via thermal energy removal, which is faster than by non-solvent exchange from the solvent, as described later [5].

#### 3.2.1. TIPS Processing

**Figure 2:**
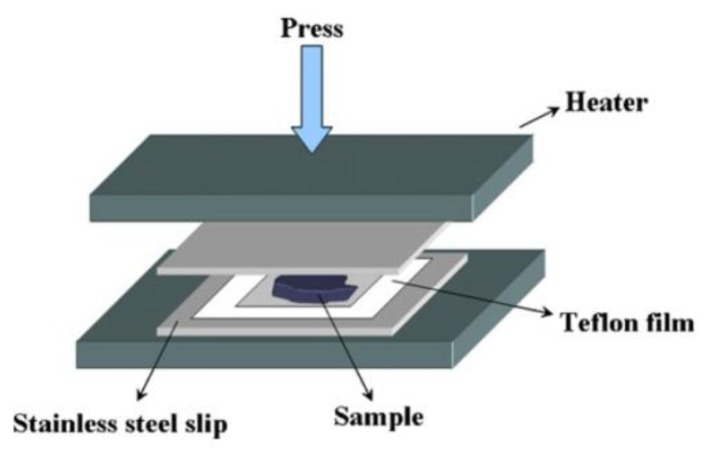
Scheme of the membrane preparation set-up via TIPS, adapted from [9].

The general procedure for TIPS is as follows. Firstly, to form a homogeneous mixture, a certain amount of a high-melting point polymer and a low molecular weight diluent (liquid or solid) mixture is placed between a pair of stainless steel plates. A Teflon film with a square opening in the center is inserted between them to adjust the membrane thickness, as shown in Figure 2. The sample is melted by heating at elevated temperature with pressure. It should be pointed out that during this step, the initial temperature (*T*_1_) must be lower than the boiling point of the diluent and is typically 25–100 °C higher than the melting temperature (*T*_m_) or the glass transition temperature (*T*_G_) of the neat polymer. Secondly, a homogeneous mixture is formed into the desired shape, which is usually a flat sheet, tube, or hollow fiber. Thirdly, a phase separation is induced by cooling at a controlled rate (thermal quenching). Then, the diluent is typically removed by solvent extraction. Finally, a micro-porous structure is produced by removing the extractant (typically by evaporation) [4]. The key point of the technology is to induce a phase separation in the membrane fabrication by removing the thermal energy of a homogeneous dope solution. Hence, the TIPS process is a balance between phase inversion path, polymer-solvent thermodynamics (interaction), cooling kinetics, extractant selection, and drying condition.

#### 3.2.2. Effect of TIPS Conditions on the Membrane Morphology

**Figure 3:**
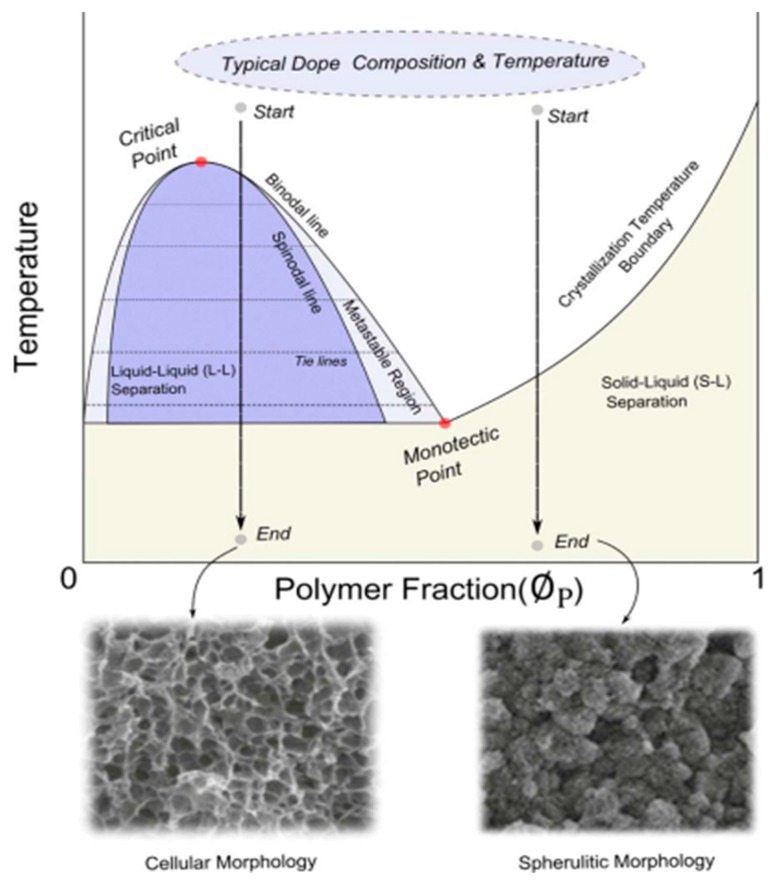
Phase diagram of the TIPS process, adapted from [63].

The TIPS process for membrane formation can be represented in terms of a temperature–composition phase diagram. As shown in Figure 3 [63,64], the membranes produced via TIPS often follow two different paths: a solid–liquid (S–L) or liquid–liquid (L–L) separation with subsequent crystallization [65]. Several investigations showed that the phase separation mechanism significantly alters the resulting membrane structure [4,5,65,66]. At the same time, the membrane performance strongly depends on the membrane pore size and porosity. As a consequence, a good control of the microporous structure is of the utmost importance in membrane fabrication [52].

Usually, when the polymer concentration is above the monotectic point (typically >30%), the dope solution goes through the crystallization temperature boundary then directly enters the solid–liquid (S–L) separation region. Conversely, the phase separation proceeds via liquid–liquid separation with subsequent crystallization at relatively lower polymer fractions. Note that if the initial polymer content in the casting solution is lower than the critical point, a continuous solvent-rich phase and a discontinuous polymer-rich phase could be formed. As a result, the final products are powders instead of a solid membrane. If a suitable polymer concentration is selected, the separation path plays a critical role on the membrane morphology [63].

Solid–liquid phase separations (S–L or L–S) are usually initiated by the formation of crystal nuclei via primary heterogeneous nucleation following stable nuclei growth through secondary nucleation. During the crystallization process, the amorphous polymer and the diluent are rejected by the growing crystals [5,67,68]. The microporous structures are then produced by removing the diluent between the lamellae or the spherulites. As discussed above, the driving force is the different polymer chemical potential in the crystalline and the solution phases [65], but crystallization thermodynamics and kinetics also play important roles for this phase separation to occur.

Liquid–liquid phase separation (L–L) is induced by the thermodynamic instability of the polymer–diluent system. The homogeneous polymer–diluent mixture is produced at a relatively high temperature and then undergoes an upper critical solution temperature (UCST)-type phase behavior by cooling the system entering an unstable region. The microstructure can be developed at the early stage of phase separation, and then the two-phase system enters the coarsening process. The kinetics continue to evolve in response to a tendency to reduce the surface energy associated with the interfacial area. This results in a reduction of the number of diluent droplets combined with an increase in droplet size [66,69,70,71,72,73,74]. The growth of diluent droplets finally stops when the polymer solution is solidified by further cooling under the crystallization temperature [66]. This means that, for semi-crystalline polymers, liquid–liquid phase separation is related to polymer crystallization, either simultaneously or subsequently [65]. Two important factors have mainly been considered to determine the relation between the final diluent droplets sizes and the membrane porosity: the phase separation temperature and the coarsening mechanism.

As mentioned above, different separation mechanisms are associated with the porous structure formation. When a specific route is selected, the final membrane pore size and pore structure strongly depend on the cooling conditions; rapid cooling provides several nuclei and leaves shorter time for crystal growth, while slow cooling provides longer times for crystal growth, resulting in membranes prepared via the L–L separation, which usually exhibit a porous, cellular-like and/or bicontinuous structure. Conversely, membranes formed via S–L separation show fuzzy spherulitic (sphere-like) structures. The parameters affecting the final membrane morphology are summarized in Table 2.

#### 3.2.3. Effect of the Polymer–Diluent Thermodynamics on the Membrane Morphology

**Figure 4:**
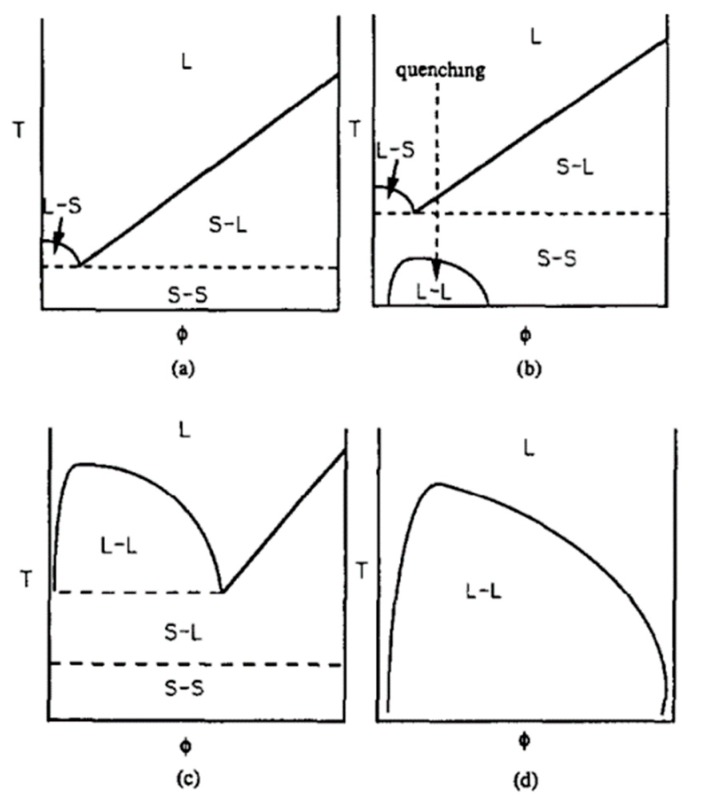
Schematic representation of the different TIPS phase diagrams, (**a**) the interaction parameter is negative and minimum, (**b**) the interaction parameter is less favorable, (**c**) the interaction parameter is high, (**d**) the interaction parameter is positive, adapted from [65].

**Figure 5:**
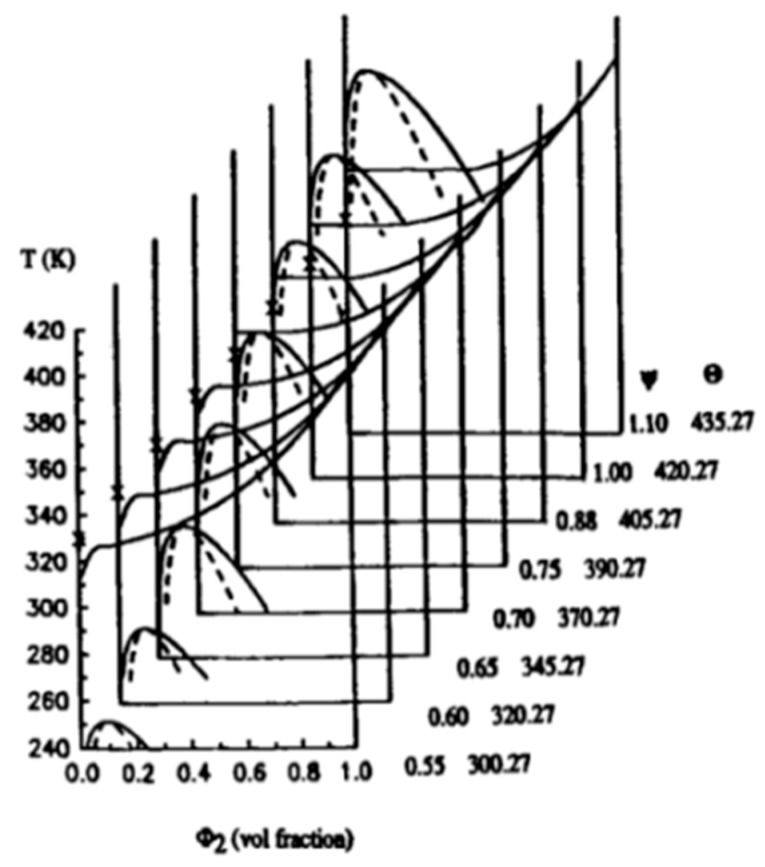
Effect of the interaction parameter (χ) on the phase diagram, adapted from [8].

For a semi-crystalline polymer–diluent system, miscibility is the main factor to determine whether a solid–liquid or liquid–liquid phase separation occurs. Miscibility can be quantified by the interaction parameter (χ) [4]. As shown in Figure 4a, the interactions between the blends are favorable when χ is negative and minimum. When cooled, the semi-crystalline polymer–diluent system has no liquid–liquid phase separation and only undergoes a solid–liquid phase separation throughout most of the composition range [65]. Figure 4b shows an unstable region below the melting point depression curve, where the interactions are less favorable (χ increasing). The blend becomes unstable, and both crystallization and liquid–liquid phase separation can take place [4,5,65,80]. According to Figure 4c, when the interactions are weak (high χ), a liquid–liquid phase separation may precede the crystallization of semi-crystalline polymers [65,81,82]. In this case, the possibility of phase separation is determined by the initial polymer concentration and the relative crystallization kinetics. Figure 4d presents a case where χ is always positive, and the amorphous phase can only occur via liquid–liquid phase separation [65,83,84,85]. This means that the phase separation process can be successfully controlled by systematically varying χ for the semi-crystalline polymer–diluent mixture. According to the Flory–Huggins lattice theory, the following relation is available [8]:

χ = ½ + Ψ θ/(T − 1)(1)

where Ψ is the entropy parameter, and θ is the theta temperature of the polymer solution. By changing the interaction parameter χ (variation of Ψ and θ separately), it is clearly observed in Figure 5 that the reduction of the L–L phase separation temperature curve is more significant than that of the melting point [8]. This may provide a situation where the L–L phase separation can occur below the L–S boundary. It is important to note that the position of the polymer–diluent mixture phase boundary is the most important factor controlling the droplet size of TIPS membranes [59].

In general, by changing the polymer–diluent composition, various interaction parameter χ can be obtained. Three methods are generally used:

- by varying the diluent types,
- by varying the components molecular weights,
- by varying the polymer concentrations.

However, for PE membranes, limitations exist for the different diluents that can be used, since only a few diluents’ molecular weight and melting points are close to PE. This results in only a few diluents being available to prepare PE membranes. The most common ones are phthalates, esters, mineral oils, normal paraffins, or isoparaffins [52,55,58]. Nevertheless, introducing a third component in the solution can change the position of the binodal line, resulting in a wide range of membrane morphology. Hence, several researchers used solvent mixtures to obtain tailor-made membrane structure and performance. However, most of these diluents are toxic (especially phthalates and esters), further limiting the range of possible options. An interesting trend for TIPS is that potential green and sustainable solvents are gaining interest to meet the increasingly stringent government regulations [86,87].

Components molecular weight is also a narrow range to choose from. For example, PE can be classified into four main classes: linear low density polyethylene (LLDPE), low density polyethylene (LDPE), high density polyethylene (HDPE), and ultra-high molecular weight polyethylene (UHMWPE). Thus, for each molecular weight range, matching diluents are required.

Table 3 summarizes the recent works related to the effect of polymer–diluent composition on the PE membrane morphology. It can be seen that, although the range of choice is narrower by varying the diluent types or varying the components molecular weight, the morphology of PE membranes is effectively controlled because the membrane fabrication process is determined by the interaction parameter χ.

**Figure 6:**
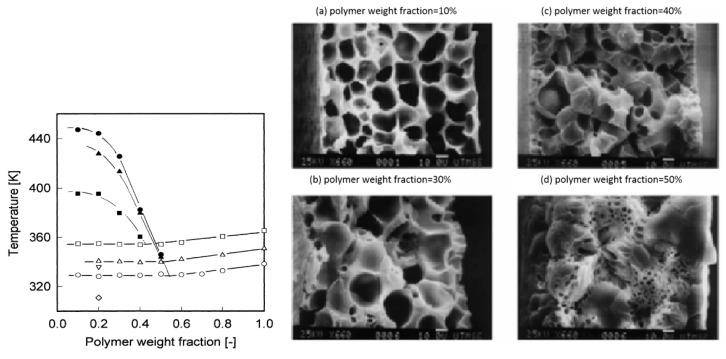
The effect of polymer concentration on the final membrane morphology. (**Left**) Dynamic phase diagrams of the polymer–diluent systems. (■) PE-DPE, (▲) Iotek7030-DPE and (●) Iotek4200-DPE. (**Right**) Micrographs of the resulting PE membranes cross-sections with a cooling rate of 10 °C/min, adapted from [66].

The third method is by controlling the polymer concentration, because the blend composition determines the phase boundaries of the system. The mixture undergoes a liquid–liquid phase separation with subsequent crystallization of the polymer at low initial polymer concentrations and solid–liquid phase separation at high initial polymer concentrations [4,65,80]. As shown in Figure 6, liquid–liquid TIPS occurs prior to crystallization for the range of 10 to 40 wt. %, while at 50 wt. %, the crystallization occurs prior to the liquid–liquid separation, and spherulites are formed. It can clearly be seen that different separation processes play a critical role in the membrane morphology. In the 10–40 wt. % range, spherical pores are well connected, and these structures are suitable for membrane separation applications. On the other hand, isolated spherical pores are produced at 50 wt. % [66].

#### 3.2.4. Effect of Phase Separation Kinetics on the Membrane Morphology

An upper critical solution temperature (UCST) is defined as a system undergoing phase separation as the temperature is lowered. As the temperature is increased, systems undergoing phase separation are said to have a lower critical solution temperature (LCST) [65]. A focus on TIPS for UCST systems is made next.

##### Solid–Liquid Phase Separation

In L–S separation, since the membrane micropores are produced by removing the diluent expelled from the growing crystal, polymer crystallization kinetics—and in some cases, that of solvent crystallization—play a major role. It can be shown that the pore size in L–S separation follows specific trends:

(I) Decreases with increasing quench temperature and cooling rate

**Figure 7:**
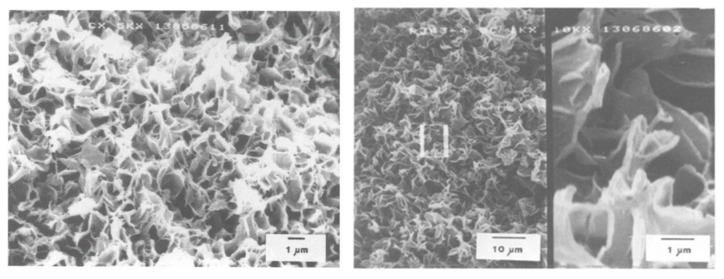
Scanning electron micrograph of the membrane structure resulting from the S–L phase separation of a 19 wt. % HDPE solution in mineral oil. The diluent was extracted with trichloroethylene (TCE) and quenched from 175 to 30 °C in water (**left**) or cooled from 175 to 30 °C at 10 °C/min (**right**), adapted from [5].

Since lower nucleation rate and longer growth period are associated with slower cooling, this allows more time for PE crystal growth without qualitatively altering the structure. Consequently, the membrane pores morphology after extraction is directly affected. Comparison of the SEM results (Figure 7) from the S–L phase separation of a 19 wt. % HDPE in mineral oil solution quenched in water via controlled cooling from 175 to 30 °C shows that the membrane morphology was clearly affected by the cooling rate. In the quenched sample, the homogeneous mixture is virtually frozen, i.e., the spherulites position is fixed, leading to the pores formed by extracting droplets densely distributed with smaller pore sizes. For slowly cooled samples, the diluent is expelled from the growing spherulites, then the diluent diffuses to the surface, leading to a local concentration gradient and some lamellae gradually crystallizing from the polymer–diluent mixture [5].

(II) Decreases with increasing polymer concentration

**Figure 8:**
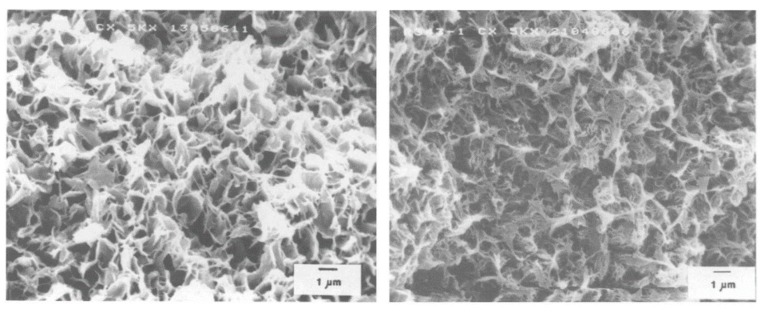
Scanning electron micrographs of the membrane structure resulting from the solid–liquid phase separation of HDPE in a mineral oil solution. The diluent was extracted with TCE from a 19 wt. % (**left**) or 34 wt. % (**right**) HDPE solution, adapted from [5].

In the highly polymer-rich case, since the initial composition is beyond the eutectic composition, the L–S phase separation is triggered by the polymer crystallization. As shown in Figure 8, pore sizes decrease with increasing polymer concentration. There are two main reasons for this phenomenon. Firstly, a change in the phase separation temperature is consistent with polymer concentration change, as reported in Figure 4. The temperature difference between the phase separation temperature and the crystallization temperature increases with increasing polymer concentration. This results in greater driving force and longer time for crystal growth. Thus, spherulite size increases as the polymer concentration increases, as less space is available for the exuded dilute droplets. Secondly, the viscosity of the polymer-rich matrix phase increases with the polymer concentration. Thus, it is more difficult for the diluent droplets to flow and aggregate. After the extraction of the diluent droplets, the membrane has a corresponding pore size distribution.

**Figure 9:**
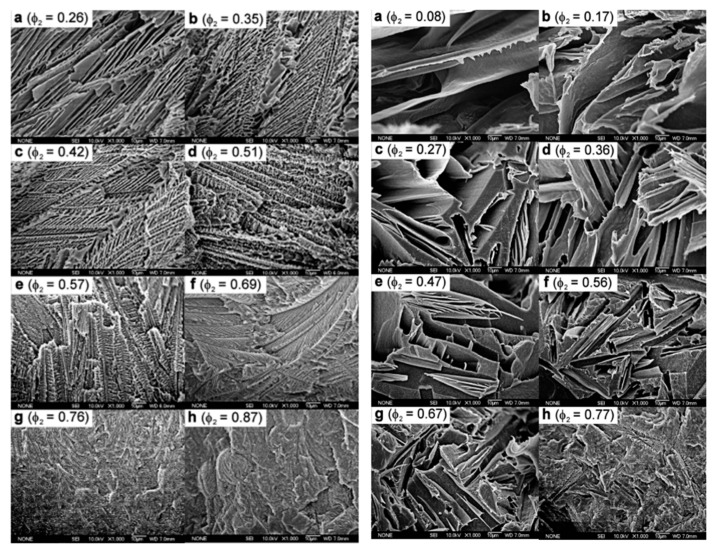
SEM micrographs showing the morphology of the fractured cross-sections of PE-diluent mixtures after extraction. Φ_2_ represents the volume fraction of LLDPE: (**left**) LLDPE/pyrene and (**right**) LLDPE/hexamethylbenzene (HMB), reprinted (adapted) with permission from [90]. Copyright (2009) American Chemical Society.

Figure 9 shows that, for some diluents, when their crystallization temperature is higher than the polymer, diluent crystallization occurs before the polymer. This phenomenon continues until the eutectic solidification begins. This induces a fixed structure—an integrally skinned porous membrane produced by extracting the diluent crystals from the solidified samples [90].

(III) Decreases with the presence of nucleating agents

Due to the extremely high nucleation density, the polymer crystals size is much smaller with the presence of a nucleating agent (heterogeneous nucleation). This substantially affects the membrane porosity in terms of higher pore numbers and smaller pore sizes [5,94].

##### Liquid–Liquid Phase Separation

**Figure 10:**
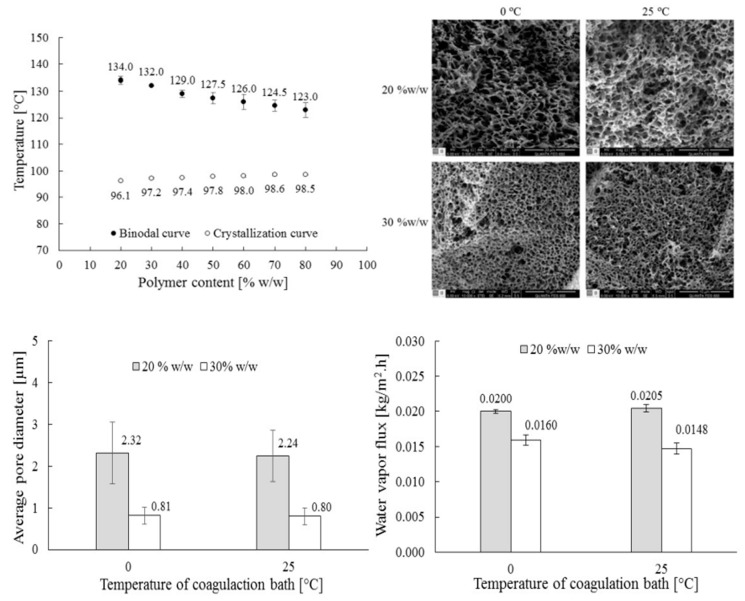
The effect of L–L phase separation kinetics on the LDPE-palm oil membrane morphology. (**Top left**) phase diagram, (**top right**) SEM images of the LDPE membrane cross-section, (**bottom left**) average pore diameter of the membrane as a function of the coagulation bath temperature, and (**bottom right**) water vapor flux through the membrane as a function of the coagulation bath temperature, adapted from [7].

As shown in Figure 10, the phase diagram of LDPE palm-oil determines the L–L phase separation prior to polymer crystallization. SEM can provide some information about the type of porous structure and how the polymer volume fraction affects the pore size, which has a high influence on the membrane performance, such as water vapor flux [7].

**Figure 11:**
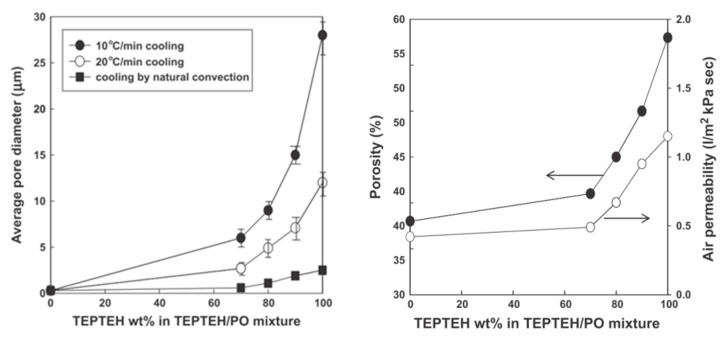
Changes in the membrane morphology and the performance when fabricated from PE//(TEPTEH/PO) = 30/70 blends as a function of triethylolpropane-tris(2-ethylhexanoate) (TEPTEH) content in the diluent mixture: (**left**) average pore diameters and (**right**) porosity and air permeability, adapted from [52].

As shown in Figure 11, the PE/(TEPTEH (triethylolpropane tris(2-ethylhexanoate))/PO) mixture exhibits a UCST type phase separation; the average pore size and the membrane porosity increase with increasing TEPTEH concentration in the diluent mixture, leading to increased air permeability [52]. Moreover, the pore size increases more when the system is slowly cooled—as opposed to fast cooling—confirming the influence of cooling kinetics.

In L–L separation, microporous structures are produced by removing the diluent droplets with an extractant. Thus, the membrane morphology is determined by the final diluent droplets’ size. A summary of the droplet growth process is described in Section 3.2.2. It can be concluded that the droplet growth rate in liquid–liquid TIPS is dependent on the polymer–diluent interfacial tension, which is influenced by several parameters as follows [4,6,52,55,56,58,59,66,69,70,71,72,73,74,83,88,95]:

- increasing with increasing volume fraction of the droplet phase,
- decreasing with increasing viscosity of the polymer-rich matrix phase,
- decreasing with decreasing the temperature difference between the phase separation temperature and the crystallization temperature,
- decreasing with increasing cooling rate.

#### 3.2.5. Effect of Extractant Selection and Drying Conditions on the Membrane Morphology

As shown in Table 4, 10 extractants were used to remove the diluent in the TIPS process to produce microporous PE membranes [89]. The membrane dimensions decreased with increasing surface tension and boiling point, leading to decreasing porosity, pore size, and permeability. Obviously, by varying the diluent and the extractant in the TIPS process, it is possible to control the membrane morphology and performance [89].

### 3.3. MSCS

In 1974, a polyolefin microporous flat membrane via melt-spinning coupled with cold stretching process was firstly reported by the Celanese Corporation. To commercialize the membranes and increase the water flux [20], changes to the membranes configuration was proposed by the Mitsubishi Rayon Corporation in 1977 [96]. The MSCS technique to fabricate hollow fiber membranes was developed, and these microporous membranes gained popularity by being used in several applications such as blood oxygenators, desalination equipments, biosensors, ultra-pure water purifiers, and membrane distillators [20,22,96,97,98].

In the MSCS process, a neat hard-elastic polymer melt is firstly spun. The precursor material must have specific properties such as high initial elastic modulus and elastic recovery [99]. In a subsequent cold-stretching step, the material is characterized by a “row nucleated crystalline lamellar” structure composed of stacked crystalline lamellae aligned parallel to the drawing direction and formed by elongational stress and then recrystallized [100]. Finally, micropores are produced by the mechanical force acting on the material and are completed during the cold-stretching step. The mechanism of micropore formation via MSCS has been reported [101,102]. It was shown that, upon stretching, the crystalline lamellae separates due to the rows of crystalline lamellae, which are in turn arranged normal to the drawing direction, and several voids are formed and interconnected. As a result, the micropores are formed in the membranes. This means that the effect of external forces is the main origin for the porous structure formation [20,22].

Compared with TIPS, the MSCS process is more difficult to control the pore size. It also requires that the precursor materials for preparing the microporous membrane are crystalline polymers such as PP [20,103,104,105], PS [106] and PE [97,98,101,107,108,109]. Furthermore, since PP has more side groups on its molecular chains, PE is a better choice in view of chemical stability and membranes properties. However, the use of diluents and extractants in the TIPS process results in waste solvent problems. Thus, the MSCS process is more environmentally friendly, since MSCS only uses a neat polymer. This also means that there is no phase separation, and its control is relatively easy [20,22].

#### 3.3.1. Effect of Temperature

In the MSCS process, the annealing temperature is an important factor affecting the micropores formation process and the pore structure of PE hollow fiber membranes [99]. It is known that the crystalline structure is reorganized when PE membranes are annealed. The aligned PE crystallinity is effectively characterized by birefringence (BR) measurements. When recrystallization occurs, more “perfect” crystalline structures are produced, and the BR increases [99,110]. Some researchers quantitatively examined the BR variation with respect to the annealing temperature [20,99]. It was shown that increasing the annealing temperature increased the BR, indicating that the micropores became more oriented. It was also found that the lamellar thickness further increased at higher annealing temperature.

**Figure 12:**
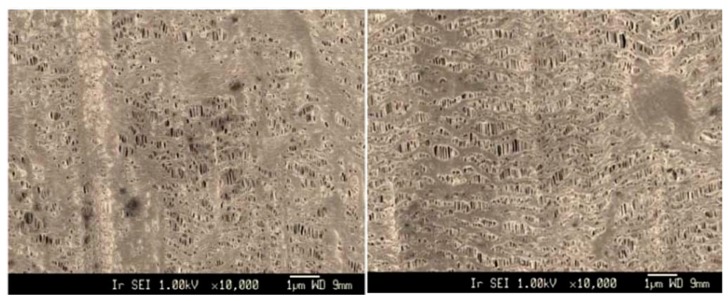
FE-SEM micrographs of HDPE microporous membranes as a function of annealing temperature: (**left**) 95 °C and (**right**) 125 °C, adapted from [99].

As shown in Figure 12, the unique microporous structures showing a slit-like shape in a pocket fashion are highly affected by the annealing temperatures. Lee et al. [99] suggested that the effect of annealing temperature on the pore structure is mainly by changing the concentration and the strength between the stressed tie molecules in the amorphous region and the crystallites lamellae. By increasing the annealing temperature, the segments of loose tie chains can be drawn into the crystallites, leading to thicker lamellae and higher overall degree of crystallinity. Elyashevich et al. [111] suggested that higher annealing temperature results in narrower length distribution of tie chains, meaning that more stretched tie chains are produced in the inter-lamellar regions. Chen et al. [110] also confirmed that the lamellae thickness increased with increasing annealing temperature up to the peak melting temperature, but higher temperature led to the formation of long-period lamellar spacing.

It is assumed that the application of an annealing temperature during the stretching process can control the final structure. Xi et al. [22] suggested that it is necessary to control the stretching process with a proper temperature in MSCS, such as the application of a two-step stretching process. In the first stretching step, the interaction between the lamellae is mainly broken under the stretching of annealed hollow fibers, and then microvoids are created on the fiber wall. Subsequently, the second stretching step results in the formation of larger micropores. In order to smoothly develop the microvoids into larger micropores, a suitable temperature below the PE melting point is the main factor to prevent microvoids collapse during the second stretching process.

**Figure 13:**
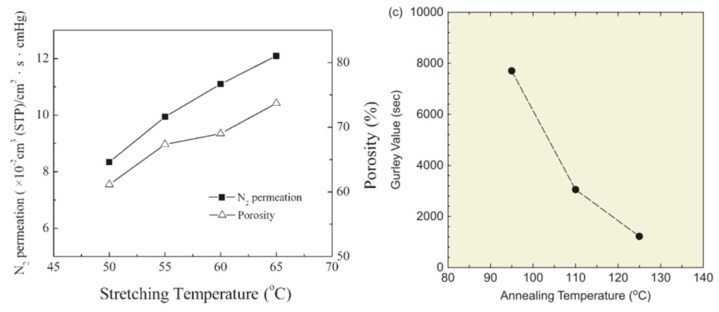
(**Left**): Effect of the stretching temperature on N_2_ permeation flux and porosity [22]. (**Right**): Effect of the annealing temperature on the Gurley value, adapted from [99].

As a next step, the morphology (porosity) and the permeability (Gurley value, N_2_ permeation) of PE hollow fibers are used to determine the performance variation by increasing the stretching temperature, as shown in Figure 13, where the Gurley value is an indicator of air permeability. Low Gurley values are associated with high air permeability. In Figure 13, the Gurley values are normalized by film thickness [112]. It is known that flexible molecular chains in the amorphous region of crystalline polymers have less resistance; therefore, molecular chains have higher mobility and orientation possibility under the applied external force and temperature. At a relatively high temperature, more molecular chains would be drawn out from the amorphous region and rearranged into fibrils along the stretching direction. Thus, higher porosity is developed by bridging the separated lamellae. This significant improvement can offer higher air permeability by optimizing the annealing temperature. This is suitable for applications such as distillation and seawater desalination [22].

In addition to the annealing temperature, the spinning temperature also affects the crystallinity and the degree of orientation of the hollow fibers. For a polymer melt, the viscosity increases with decreasing temperature, which is necessary to increase the stress in the spinning process, leading to a higher level of orientation in the fibers. Therefore, hollow fibers spun at higher temperature have lower birefringence, higher bubble point pressure, smaller pore size, and more uniform pore distribution [20].

#### 3.3.2. Effect of the Stretching Ratio

The degree of melt extension can be described by the stretching ratio (*R* = *V*_2_/*V*_1_) defined as the ratio between the extrusion speed (*V*_1_) and the take-up speed (*V*_2_). This parameter mainly governs the morphology in terms of pore size, distribution, orientation, geometry, etc.

The stretching ratio has been reported to affect the stress along the fiber direction [99,102]. For example, increasing the stretching ratio from 1.35 to 2.28 led to more elongated fibrils, and the space between the separated lamellar crystals became larger [22]. Also, increasing the stretching ratio from 2.3 to 3.3 led to more uniform pore size distributions, and the amount of pores (diameter < 1 µm) rapidly increased to become the main part of the pore size distribution. Several theories were proposed to explain these results [113,114]. Firstly, from a position originally perpendicular to the fiber direction, the lamellae rotated to a position parallel to the fiber direction. Secondly, molecular chains in the lamellae were oriented, and then the microvoids linked by fibrils were formed. Thus, higher stretching ratio means that more fibrils bridging the separated crystalline lamellae are developed. Therefore, microvoids or micropores continuously grow in size. However, there is a critical stretching ratio for which the fibrils in the microvoids break, resulting in the formation of cracks and leading to poor structures [22].

By increasing the stretching ratio within a reasonable range, higher porosity and permeability of PE microporous hollow fiber membranes can be produced. Experimental results confirmed that the relationship between the stretching ratio, the porosity, and the N_2_ permeation of PE membranes is directly correlated to the stress range. When the ratio increased from 100 to 170%, the N_2_ permeation increased by 31%, while the porosity increased by 13% [102].

#### 3.3.3. Effect of the Stretching Rate

To get a better understanding of the stretching rate effect on the PE hollow fiber membrane structure and properties, the mechanism of resistance to stress cracking is used. During the stretching process, the formation of fibrils recovered in the PE hollow fiber wall and break-up in the hollow fiber wall occur at the same time. With an increased stretching rate, more fibrils are formed and destroyed. As a result, the amount of smaller size pore (average pore size < 1 µm) increases. As shown in Table 5, under a relatively high stretching rate, the average pore sizes slightly decreases, and the pore size distribution gets more uniform, and the N_2_ permeation and the porosity further increase with a more uniform porous structure, leading to better performance of the PE hollow fiber membranes with increased stretching rate [22].

#### 3.3.4. Effect of Cooling Ways

Luo et al. [21] prepared hollow fiber membranes by MSCS and selected air, water, and di-isononyl-cyclohexane-1,2-dicarb-oxylate (DINCH) as the cooling media to investigate the effect of various cooling methods and cooling rates on the membrane morphology. The results showed that when water and DINCH were used as the heat transfer medium, a layer of amorphous phase and fewer uniform pores—which only had a small part of the slit-shaped pores—were obtained on the surface, because the fast cooling rate froze the surface molecules and decreased the crystallinity and orientation. However, when air was used as the cooling medium, higher crystallinity and better orientation of the annealed hollow fibers were obtained, mainly due to the slow cooling rate. As a result, hollow fiber membranes prepared by slow cooling had better pore interconnectivity and performances [21].

As demonstrated above, although several parameters must be controlled in MSCS, the key factors significantly influencing the membrane morphology can be classified into temperature, stretching ratio, stretching rate, and cooling conditions. The stretching ratio and the stretching rate mainly govern the crystalline lamellae alignment, while the stretching temperature and the cooling conditions significantly influence the number of stretched tie chains and fibrils bridging the narrow cracks [22,99,102,103,110]. It is possible to control these four key parameters inside an optimum range during the stretching process to improve the porous structure and the overall performances of hollow fiber membranes. For higher membrane porosity and more uniform pore distribution, the following strategies can be used:

- increasing the annealing temperature within the peak melting temperature range,
- increasing the stretching ratio within a reasonable range,
- increasing the stretching rate below a critical value (membrane break-up),
- decreasing the cooling rate.

## 4. PDMS Membranes

Based on the outstanding advantages of PDMS, such as non-toxicity, high hydrophobicity, chemical resistance, gas permeability, optical transparency, environmental friendliness that does not bio-accumulate, flexibility, low costs, and good molding capability [34,115], through-hole PDMS membranes found wide applications in the biomedical and chemical fields, such as sterile filtration, cell sorting, biomolecular separation, termed organs-on-a-chip systems, microfluidic devices, thin film extraction, lab-on-a-chip devices, micro total analysis systems, and permeation passive samplers [34,115,116,117,118].

A number of methods have been reported to fabricate PDMS through-hole membranes [33,34,119,120]. A focus on micromolding, imprinting/soft molding, manual punching, and the novel three-dimensional (3D) printing technique is presented here.

### 4.1. Properties of PDMS Membranes

**Figure 14:**
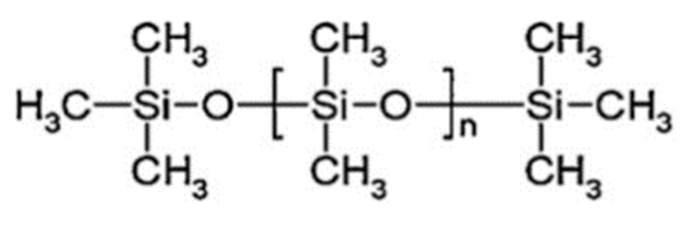
The structure of PDMS (CAS number 63148-62-9), adapted from [115].

PDMS is made of a flexible (Si–O) backbone and a repeating (Si(CH_3_)_2_O) unit (see Figure 14). For PDMS, the molecular weight varies from 1 × 10^4^ to 6 × 10^4^ gmol^−1^ due to the amount of (Si(CH_3_)_2_O) repeating units. Consequently, several material properties, such as viscoelasticity, are affected [121]. Furthermore, these viscoelastic properties can be modified by cross-linking (vinyl cross-linking) [122] or by filler addition (silicon dioxide) to the polymer network to meet the requirements of the current applications [123]. Table 6 lists some typical PDMS properties, especially because it is hydrophobic and translucent [115,124]. Today, PDMS is widely used in nanomembranes because of its remarkable advantages, such as chemical resistance, mechanical properties, gas permeability, optical transparency and biocompatibility, as well as its high molding capacity, leading to the production of nanomembranes and micro/nanofluidic systems [34,37,120,125].

### 4.2. PSμM

#### 4.2.1. PSµM Processing

**Figure 15:**
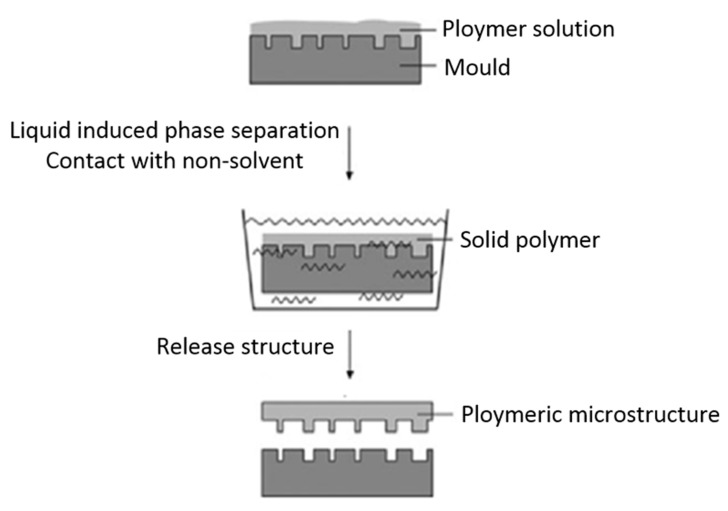
Schematic representation of the PSμM by the wet method, adapted from [126].

A novel micromolding process based on polymer phase separation has received increasing attention in recent years, and Figure 15 presents a schematic representation of this method. The main process of spin-coating on molds consists of four steps as follows [32,33,34]:

1. Firstly, fabrication of dedicated patterning systems occurs. In most case, an additional coating of anti-sticking layers on the molds prior to the injection of the pre-polymer is needed to release the nano-membrane from the mold.
2. Subsequently, there is prepation of a PDMS solution with a specific composition, and then the solution is spin-coated over the fabricated molds.
3. To open the through-holes, the cured PDMS membranes are further processed via etching or thermal compression.
4. Finally, remove the photoresist posts to expose the through-holes.

Although various methods such as conventional photolithography processes (soft-lithography), electron-beam lithography [127], laser interference lithography [128], or focused ion-beam milling [129,130] can be used to pattern the nano-columns in step 1, the difficulty in the fabrication of patterned columns (SU-8) at sub-μm diameter ranges limits the use of PDMS membranes for nanometer-sized through-holes. Furthermore, patterning such molds is labor intensive and requires dedicated patterning systems, which are expensive [34].

**Figure 16:**
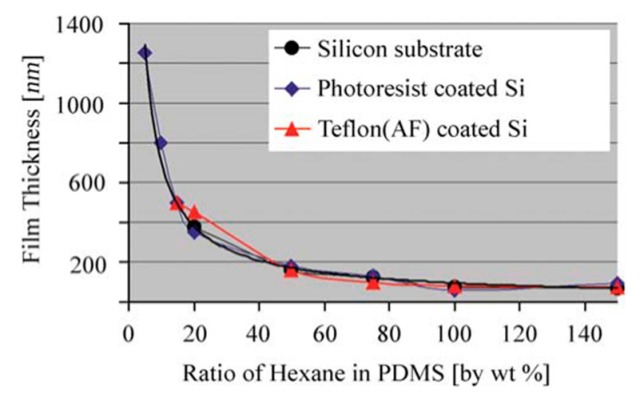
PDMS film thickness on three different substrates with different dilution ratios in hexane, adapted from [131].

For step 2, the solution composition and concentration play critical roles in the PDMS membrane morphology. Thangawng et al. [131] reported on the effect of different PDMS ratios in hexane on the membrane thickness. The spin rate and the duration were kept constant at 6000 RPM and 150 s, respectively, but they used three different substrates: silicon, a Si coated photoresist, and a Teflon (AF) coated Si. The results in Figure 16 show that the membrane thickness was independent of the substrate types. However, the thickness decreased with an increasing amount of hexane in the solution. Lötters et al. [132] obtained similar results by showing that the membrane thickness could only be reduced down to 3 μm if the PDMS was not diluted with a curing agent. Thus, in most samples, a PDMS pre-polymer:curing agent ratio of 10:1 was used to achieve highy flexible and ultra-thin PDMS membranes.

For step 3, the first task is to select a method to open the through-holes and cure the membrane. This can be done via dry etching, plasma etching, chemical etching, thermal compression, and blowing. The processing parameters (time, pressure, and power) must be controlled to obtain the tailor-made PDMS membranes.

For step 4, the through-hole PDMS membrane can be obtained by demolding the cured PDMS layer from the mold using chemical reagent and completely dissolving the sacrificial photoresist (PR) structures [34]. Another method for a perforated membrane is to directly peel off the membrane from the molds [33,133].

#### 4.2.2. Effect of Processing Parameters on Membrane Morphology

##### Etching Time

**Figure 17:**
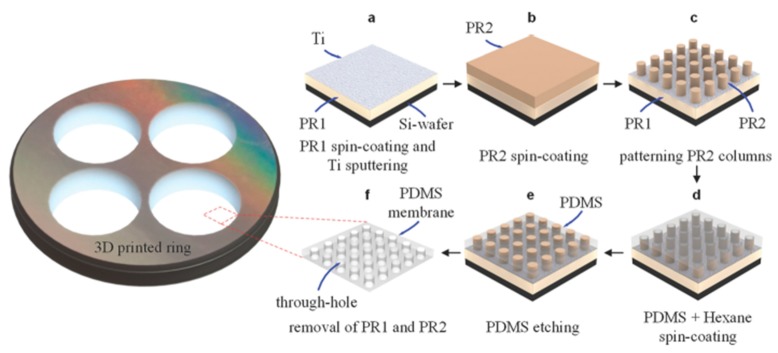
Fabrication process of a free-standing and sub-µm thick PDMS through-hole membrane. Photoresist (PR) layer (OiR 907-17i, Fujifilm, Japan) of 1.71 ± 0.04 µm in thickness is used as the sacrificial layer. Si wafer (525 µm thick, Okmetic, Finland) is used as a support base, adapted from [34].

**Figure 18:**
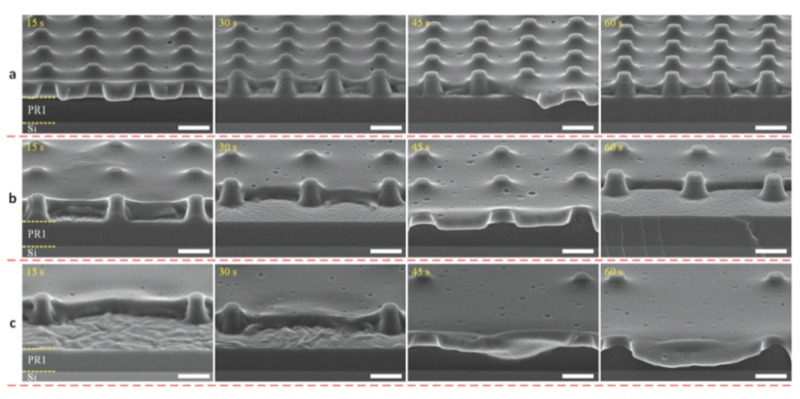
Cross-section HR-SEM images of the PDMS-coated PR column arrays with different pitches of: (**a**) 3 µm, (**b**) 5 µm, and (**c**) 10 µm with various etching times, adapted from [34].

Tibbe et al. [34] used the soft-lithography method to fabricate sacrificial PR arrays with different hole interspacings of 3, 5, and 10 μm as molds. Secondly, a 1:10 PDMS:hexane solution was spin-coated over the fabricated PR column arrays at 6000 RPM for 3 min and subsequently left to cure. As a result, a PDMS layer thinner than the column height was produced. Thirdly, reactive plasma etching of the PDMS membrane was conducted in a reactive ion etching system by precisly controlling the etching time and the etching rate by tuning the amount of gas mixture and power. Finally, the PDMS through-hole membrane was released in acetone using a 3D-printed ring as a support. By doing so, they sucessfully prepared free-standing PDMS membranes at various sub-μm thicknesses down to 600 ± 20 nm with nanometer-sized through-hole (810 ± 20 nm diameter) and over areas as large as 3 cm in diameter. The whole fabrication process is described in Figure 17. During the membrane fabrication, the researchers mainly investigated the etching time as the main parameter, which has a significant effect on the membrane morphology. Figure 18 presents the evolution of the PDMS through-hole membranes etched for various times. The average thicknesses of the PDMS through-hole membranes at 0, 15, 30, 45, and 60 s were 1345, 1170, 940, 749, and 600 nm, respectively. By increasing the etching time, the membrane thickness was thinned down to sub-μm dimensions. In addition, when spin-coating a PDMS solution over a patterned nano-column array, some unexpected breaking of the PDMS thin film is typical [134,135]. It can be concluded that the ductility of the PDMS thin films is important and related to the intrinsic flexibility of the PDMS selected.

##### Effect of the Power, Oxygen Partial Pressure, and Etching Time

Jo et al. [32] reported a robust and simple method for thin PDMS membrane. Firstly, the mold master was formed on a silicon wafer using an epoxy-based photoresist (SU 8) and standard photolithography procedures. Secondly, a curing agent and PDMS prepolymer (Sylgard 184 Silicone Elastomer Kit, Dow Corning) were thoroughly mixed in a 1:10 weight ratio, and the PDMS prepolymer mixture was poured onto the master mold. Then, a transparency film was placed over the poured prepolymer mixture. Next, a multilayer stack of aluminum plates, the mold master, the PDMS prepolymer mixture, a transparency film, a rigid Pyrex wafer, and a rubber sheet were used to form a compression mold. All the layers were clamped tightly during the cure. Then, the individual components were bonded together with a reactive ion etching (RIE) system. Finally, the thin PDMS membranes were peeled off from the master mold after curing. To determine the optimum conditions, the experiments studied the effect of three processing parameters: power, oxygen partial pressure, and etching time. The results showed that low power and short etching time were better than high RF power and long etching time. The optimum pressure range was 75–120 mTorr [32].

##### Effect of Gas Stream Pressure

Kang et al. [135] reported a simple method using spin-coating of the PDMS pre-polymer on the Si wafer followed by gas blowing followed by a recovery process to obtain well-defined, flat PDMS membranes. This method can make well-defined holes in a PDMS membrane prepared at both high and low RPM. Furthermore, it can be repeatedly and selectively used, even with the PR mold fabricated by the single-step lithography as well as two-step lithography. The air-blowing process through the nozzle was maintained for several seconds on the SU-8 patterns to remove the PDMS prepolymer from the pattern. The results showed that 8–15 kPa was the optimum pressure range. For air pressures above 15 kPa, damages on the PDMS surface for 40 μm thickness membranes were observed, while pressures below 8 kPa were too low to blow off the residual prepolymer from the PR post.

**Figure 19:**
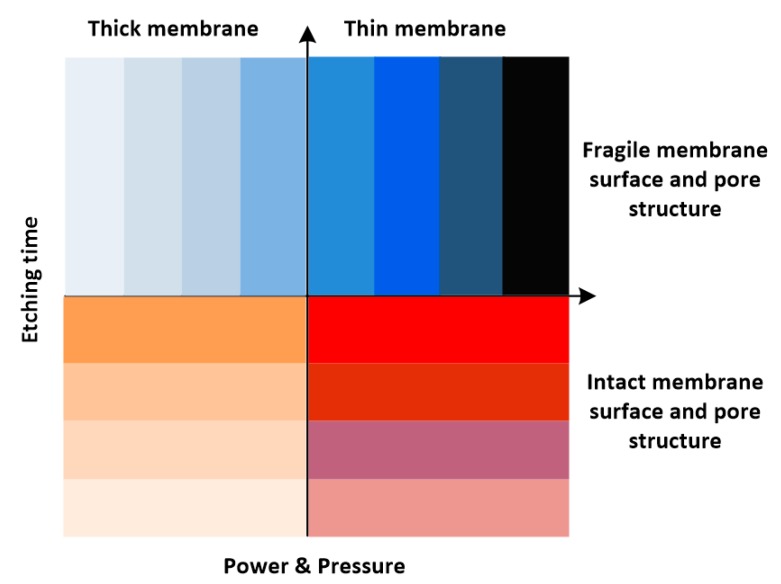
Schematic representation of the different parameters controlling the PDMS membrane morphology via PSµM.

As presented above, the key parameters controlling the structure include etching time, power, and pressure. By increasing the etching time, the membrane thickness decreased. Low power and the pressure within an optimum range were better than high power with very high or very low pressure. For a clearer understanding, Figure 19 presents a schematic representation of the different factors that control the PDMS membrane morphology via PSµM.

### 4.3. Imprinting/Soft Molding

**Figure 20:**
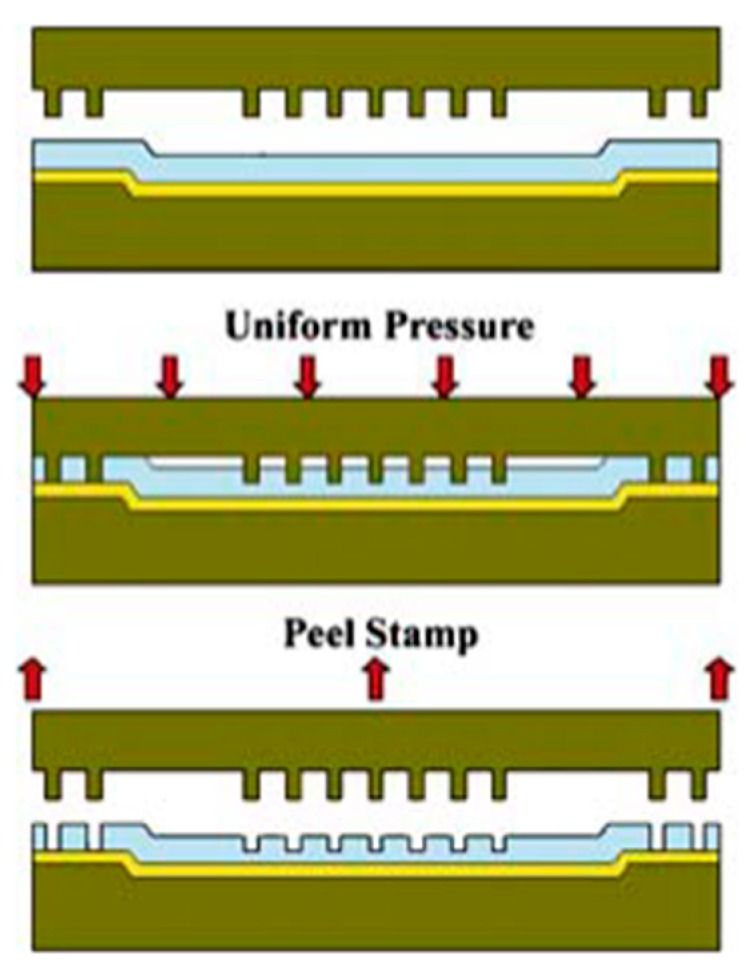
Schematic representation of the PDMS through-hole membrane fabrication steps via imprinting/soft molding, adapted from [131].

A technique of transferring imprinting pattern is called soft molding [35,36]. Figure 20 presents the process of through-hole pattern on a flat PDMS membrane via imprinting/soft molding. Firstly, the PDMS solution was spin-coated to a specific thickness on a substrate with an adhesion reduction layer. Then, a selected mold was carefully placed on the PDMS membrane with an appropriate pressure to displace and penetrate through the uncured PDMS membrane to form the desired patterns. Susquently, the membrane with the mold was fully cured while maintaining the load, hence a desired perforated membrane was sucessfully produced [131]. It is worth mentioning that the volume shrinkage of the photopolymerizable polymer used for imprinting during the photopolymerization was different, resulting in the mold being easily released after imprinting, hence a polymeric membrane with the desired through-holes was obtained [119,136].

The imprinting process consists of pressing a positive pillar mold into a pre-polymer layer coated on a flat surface. Based on the formation process of through-hole PDMS membranes, some researchers reported that the main parameters include the imprinting pressure and the drying method [119,131,136,137]. However, this method is not suitable to produce uniform PDMS nanomembranes over large areas due to increased pre-polymer viscosity, which leads to the difficulty of achieving the required pressure to produce the complete through-holes. Moreover, excessive pressure leads to higher production costs [34].

### 4.4. Manual Punching

Heo et al. [37] used the method of manual punching to produce through-hole PDMS membranes. They prepared the casting prepolymer (Sylgard 184, Dow-Corning) at a 1:10 curing agent-to-base ratio. The prepolymer was then poured on the positive side of SU-8 (MicroChem) using the backside diffused-light photolithography method to produce the required features. Subsequently, the membrane was cured at 60 °C for 60 min, and the holes were punched in by a sharpened 14-gauge blunt needle.

A manual punching process is merely suitable for low yield patterning PDMS through-hole nanomembranes over small footprints. One reason is the process of punching a needle through a continuous PDMS membrane is time consuming. Another reason is the rather difficult membrane handling at the nanoscale for the punching process [34].

### 4.5. D Printing Technique

**Figure 21:**
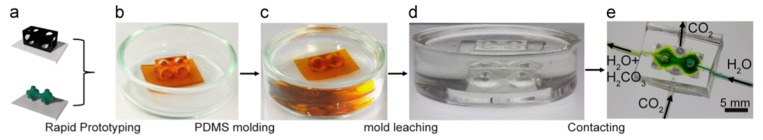
Step-by-step fabrication of a tricontinuous PDMS membrane based on Schwarz-P geometry, adapted from [38].

Femmer et al. [38] developed a new sacrificial lithography technique to produce three-dimensional membrane geometries using rapid prototyping (Figure 21). The approach is to print an acrylate-based sacrificial negative mold and use it as a template for the membrane fabrication. Based on “triply periodic minimal surfaces” (TPMS) structures, the molds were designed as Schwarz-D, Schoen-G, and Schoen-P. The molds were then immersed in a PDMS prepolymer formulation (Sylgard 184, silicone/crosslinker 7:1), degassed in vacuum, and subsequently cured at 65 °C for 30 min. Later, the molds were treated with 1 M NaOH at 70 °C. Finally, 1 mm thick micro-structured TPMS-PDMS membranes were obtained, which were thick enough to withstand pressure differences of 2 bar. It was shown that these membranes had 30–60% higher CO_2_ diffusion coefficients than common hollow-fiber membranes with similar dimensions.

An alternative approach was proposed by printing a PDMS membrane using a direct light processing (DLP) printer to print silicone structures. For optimum compatibilization, the silicone and the photoinitiator were mixed in tetrahydrofuran and then removed in vacuum. The results showed that the printed PDMS membranes had similar selectivity performance as standard PDMS membranes. Conversely, 15% lower permeability than common membranes was related to their larger thickness (840 µm) [39].

Gernally, because of the high cost and the limited hardware available for printing, limited studies have been published on obtaining nanometer resolution in 3D builds to produce perforated PDMS membranes. However, the 3D printing technique could allow an unprecedented control over membrane morphology by allowing both the micro- and the macro-structure of the membrane to be designed and produced in one step, even allowing membrane module fabrication to be controlled in a single machine/process from membrane material to membrane module. Thus, the use of 3D printing in membrane fabrication is a promising method for the near future [138].

## 5. PP, PI, and PTFE Membranes

### 5.1. Properties and Applications of PP, PI, and PTFE

Besides PE, PDMS, PSU, and PVDF membranes, numerous studies reported the developments and the applications of other commonly used microporous polymeric membranes such as PP, PI, and PTFE. Table 7 lists the composition and the properties of these polymers.

Microporous polymeric membranes based on PP, PI, and PTFE have been widely used in several fields due to their outstanding properties and low cost. The main areas include both hydrophilic and hydrophobic applications. For hydrophilic applications, water treatment and reuse [143], biochemical and biomedical applications [144,145], battery separators [146,147], and chemical valve applications [148,149,150,151] were developed. However, fouling is the main challenge for hydrophilic microporous polymeric membranes with high performance applications [152], but hydrophilic modification can improve the fouling-resistant properties of polymeric membrane [153]. For hydrophilic applications, membrane distillation and emulsification [154,155,156,157,158] as well as membrane gas absorption [159,160,161] and membrane crystallization [162,163] are available, but other applications such as solvent extraction [164], water-oil separation [165] and vegetable oil membrane filtration [166,167] are investigated. However, it is necessary to prepare super-hydrophobic polymeric membranes with appropriate pore size to deal with the wetting issues and obtain long term performance [153].

### 5.2. Preparation Methods

To fabricate membranes using PP, PI, and PTFE, the most common methods are TIPS, stretching, electrospinning, NIPS, and a new method that was recently developed, i.e., auxiliary-assisted pore forming. Typical properties of these methods are compared in Table 8. It is important to note that the final polymer membrane morphology and performance are optimized by the combined effects of all the processing parameters.

## 6. Conclusions and Recommendations

The development of porous polymeric membrane is an important research application in separation technology. This article reviewed the development of porous polymeric membranes from the perspectives of membrane materials and fabrication methods. Polymers such as PE, PDMS, PP, PI, and PTFE were reviewed due to their outstanding thermal stability, chemical resistance, mechanical strength, and low cost. Different fabrication methods such as TIPS, MSCS, PSµM, imprinting/soft molding, manual punching, and three-dimensional printing were also presented and discussed in terms of the strategy to produce porous polymeric membranes with a controlled membrane morphology and performance via key factors associated with each processing method. This included the system, the solution, the processing, and the ambient parameters/conditions. Nevertheless, some challenges still remain, which are subject for future innovation. For example, better materials (properties and stabilities), better fabrication processes (simpler and cost efficient), and applications (new areas) are of interest. Investigations should also aim at improving our understanding of the fouling and the wetting mechanisms, as well as the transport phenomena related to the membrane preparation and use to further accelerate the progress in membrane technology, especially for long term properties.

## Figures and Tables

**Table 1:** Various preparation methods for porous polymeric membranes.

Method	Pore Formation Mechanism	Advantages	Disadvantages	Refs.
TIPS	The sites occupied by the diluent become micropores after their removal.	1. Suitable for various polymers, especially for semi-crystalline polymers that cannot be easily dissolved by solvents. 2. Membranes are inherently reproducible and less prone to defects than other phase inversion methods.	1. Low mutual affinity between the solvent and the non-solvent, resulting in the surface pore hardly being tuned. 2. Expensive and the organic solvents used are usually not environmentally friendly.	[4,5,6,7,8,9]
NIPS	Resulting from liquid–liquid phase demixing.	NIPS can effectively control the pore size and other surface characteristics of the membranes with the help of additives.	Difficult to precisely control the phase inversion process.	[10,11,12,13,14,15]
VIPS	Resulting from the transfer at the interface, non-solvent (gas) inflow and solvent outflow.	VIPS enables modifying and tailoring both flat-sheet and hollow-fiber polymer membrane morphologies.	The development of commercial polymer membranes still remains limited.	[10,16,17,18,19]
MSCS	Resulting from mechanical forces acting on the membranes in a subsequent cold-stretching step.	1. Simple and economical process, suitable for large scale fabrication. 2. No solvent, diluents, or additives in process. 3. Extremely high mechanical strength.	Highly oriented membrane structure results in low tear resistance in the transverse direction.	[20,21,22]
Electro-spinning	Resulting from the evaporation of the diluent.	Directly produce superhydrophobic polymer membranes and highly porous structures of smooth non-woven nanofibers, it is simple, inexpensive, and high productivity.	Limited production capacity and low reproducibility.	[10,23,24,25,26]
Track etching	Irradiation produces tracks in the foils and pore formation via chemical etching.	The membrane pore size, shape, and density can be precisely determined in a controllable manner.	Limited for some particular uses and large-scale applications. It is also highly cost extensive.	[10,27,28,29]
Sintering	Resulting from the sintering transformation driven by high temperatures.	Widely used in the commercial production of inorganic membranes and some polymer membranes.	Sintering is costly, processing has materials limitation, material synthesis, and phase stability.	[10,30,31]
PSµM	Resulting from the polymer phase separation.	The method can fabricate a structure in the sub-micrometer range and can prepare a surface with two-tier hierarchical structures featuring a super-hydrophobic property.	Limited processing/production capacity.	[32,33,34]
Imprinting/soft molding	Resulting from the molding with an appropriate pressure.	The method can produce uniform porous membranes with a desired through-hole pattern.	The method is not suitable for fabricating polymeric nano-membranes over large areas.	[35,36]
Manual punching	Resulting from the combination of backside diffused-light photolithography and needle punching.	The method can fabricate polymeric nano-membranes with uniform relief features.	The method is merely suitable for low yield patterning through-hole membranes over small footprints.	[37]
3D printing technique	Resulting from printing an acrylate-based sacrificial negative mold and using it as a template.	Possible to create almost any geometrically complex shape or feature in a range of materials across different scales.	1. The technique is currently in its infancy, thus limited resolution and printing materials are available, 2. The technique has high cost.	[38,39]

TIPS = thermally induced phase separation, NIPS = non-solvent induced phase separation, VIPS = vapor-induced phase separation, MSCS = melt-spinning combined with cold-stretching, PSµM = phase separation micromolding.

**Table 2:** Effect of TIPS conditions on the membrane morphology.

Separation Route	Cooling Conditions	Description of Membrane Morphology	Refs.
S–L	rapid cooling	Yield small spherulites with small pore sizes and high mechanical strength.	[63,75,76,77,78]
	slow cooling	Yield larger fuzzy spherulitic structures.	
L–L with subsequent crystallization	long time period in the L–L region	Yield larger pores with a cellular structure.	[79]
	short time period in the L–L region	Yield smaller pores with a porous cellular-like and/or bicontinuous structures.	[75,79]

**Table 3:** Recent investigations on the effect of polymer–diluent composition on PE membrane morphology.

PE	Diluent	Extractant	Membrane Morphology		Controlled Parameters	Ref.
HDPE	DOP/isoparaffin		Pore size	PE/DOP: 0.17 μm	Diluent mixing ratio.	[88]
				PE/isoparaffin: 0.07 μm		
				PE/(DOP/isoparaffin): 0.07–0.5 µm		
HDPE	paraffin/PTMG	acetone	Pore size	0.2–12 μm	Paraffin/PTMG mixing ratio and molecular weight of PTMG.	[59]
			Porosity	34–57%		
PE	mineral oil	Trichloroethylene	Porosity	5.9–53%	Pore dimensions increased by decreasing the surface tension and boiling point of the extractant.	[89]
PE	LP	ethanol	Pore sizes	3–5 µm	Pore size and porosity increased with higher quenching temperature and longer annealing time.	[57]
			Porosity	50–60%		
			Thickness	100 µm		
HDPE	1. DIDP 2. LP	ethanol/DIDP, hexane/LP	Structure	Asymmetric; hollow fiber	Pore size controlled by diluent type, increasing with higher molecular weight, shorter air gap distance and higher bath temperature.	[6]
			Thickness	160 µm		
			Pore size	HDPE25/DIDP: 0.35–0.40 µm		
				HDPE13/DIDP: 0.23 µm		
				HDPE25/LP: -		
	1. Pyrene 2. HMB	methanol for pyrene, acetone for HMB	Structure	LLDPE/pyrene: locally aligned layers	Microporous structure can be controlled by the type of the crystallizable diluents as well as the composition of the mixtures.	[90]
				LLDPE/HMB: plate-like		
			Pore size	LLDPE/HMB > LLDPE/pyrene		
LDPE	Palm oil	hexane	Pore size	0.8–2.3 µm	Increasing the polymer content decreases the pore size and water vapor permeability.	[7]
			Thickness	≤400 µm		
HDPE	1. TEPTEH 2. paraffin oil	ethanol	Thickness	300 µm	Diluent composition and its mixing ratio	[52]
			Pore size	PE/TEPTEH: 2.5 µm		
				PE/PO: 0.3 µm		
				PE//(TEPTEH/PO): 0.3–2.5 µm		
			Porosity	PE/TEPTEH: 42.5%		
				PE/PO: 32%		
				PE//(TEPTEH/PO): 32–42.5%		
HDPE	1. SBO 2. DOP	acetone	Pore size	PE/DOP: 0.2 µm	Pore size increases with increasing phase separation temperature and by controlling the diluent mixture.	[55]
				PE/SBO: 0.6 µm		
				PE/SBO/DOP: 1.2 µm		
HDPE	EVA	xylene	Structure	PE: EVA (60:40): interpenetrating network	Blend composition, final process temperature, and cooling rate.	[91]
				PE: EVA (50:50): co-continuous		
				PE: EVA (30:70): domain/matrix		
HDPE	PP/clay platelets/SEPS		Structure	asymmetric and co-continuous	Two melt blending methods and diluent-mixture.	[92]
			Pore size	PP/HDPE: 6.46 µm		
				PP/HDPE/SEPS: 3.82 µm		
				PP/HDPE/SEPS/clay: 2.02–2.96 µm		
LLDPE	OPE/p-xylene		Structure	PE: smooth surface	Viscosity (increasing OPE led to viscosity reduction of the blends).	[93]
				PE/OPE: fibrous		
HDPE	PE-b-PEG/DPE	ethanol	Thickness	100 µm	Mixing ratio of PE-b-PEG.	[9]
			Pore size	HDPE/DPE: 1.32 μm		
				HDPE/DPE/PE-b-PEG (5 wt.%): 2.25 μm		
				HDPE/DPE/PE-b-PEG (10 wt.%): 2.67 μm		
				HDPE/DPE/PE-b-PEG (20 wt.%): 3.80 μm		

HDPE = high density polyethylene, LDPE = low density polyethylene, LLDPE = linear low density polyethylene, DOP = dioctyl phthalate, PTMG = polytetramethyleneglycol, DIDP = diisodecyl phthalate, LP = liquid paraffin, HMB = hexamethylbenzene, TEPTEH = triethylolpropanetris (2-ethylhexanoate), SBO = soybean oil, EVA = ethylene vinyl acetate, PP = polypropylene, SEPS = polystyrene-block-poly(ethylene-ran-propylene)-block-polystyrene, OPE = oxidized polyethylene, PE-b-PEG = polyethylene-block-poly(ethylene glycol), DPE = di-phenylether.

**Table 4:** Membrane dimensions using 10 extractants to remove the diluent and produce microporous PE membranes [89].

Component	Solubility Parameter (MPa^1/2^)		Boiling Point (K)	Surface Tension at 298 K (mN/m)	Dimension in the Extrusion Direction (cm)	Dimension in the Perpendicular Direction (cm)	Thickness (µm)	Porosity (%)
	Total	Dispersive						
PE	16.2	16.2	-	-	-	-	-	-
Trichloroethylene	18.7	11.7	360	28.70	4.44	3.60	210	24.0
p-Xylene	18.1	16.5	411	28.01	4.02	3.00	195	5.9
Toluene	18.3	16.4	383	27.93	4.32	3.20	200	17.0
Tetrahydrofuran	18.5	13.3	339	26.40	4.86	3.40	217.5	40.0
Cyclohexane	16.8	16.4	354	24.65	4.80	3.40	217.5	39.0
2-Butanone	19.3	14.1	353	23.97	5.04	3.52	222.5	40.0
Ethyl acetate	18.2	13.4	350	23.39	5.16	3.52	225	48.0
Heptane	15.3	15.3	371	19.65	4.98	3.52	225	45.0
Hexane	14.9	14.9	342	17.89	5.34	3.68	232.5	50.0
Pentane	14.4	14.4	308	15.49	5.46	3.76	240	53.0

**Table 5:** Properties of microporous hollow fibers produced using different stretching rates [22].

Stretching Rate (mm/min)	Porosity (%)	Average Pore Size (µm)	N_2_ Permeation (cm^3^(STP)/cm^2^∙s∙cmHg)
200	70	0.62	4.96 × 10^−2^
280	75	0.58	7.05 × 10^−2^
320	80	0.55	7.09 × 10^−2^
360	81	0.57	8.10 × 10^−2^
400	82	0.50	10.00 × 10^−2^

**Table 6:** Typical properties of polydimethylsiloxane (PDMS).

Properties	PDMS
Density (kgm^−3^)	965
Melting point (°C)	N/A
Glass transition temperature (°C)	−127
Elastic modulus (kPa)	250
Dielectric constant	2.72–2.75

**Table 7:** The composition and properties of polypropylene (PP), polyimide (PI), and polytetrafluoroethylene (PTFE).

Polymer	Composition	Properties	Refs.
PP	–CH_2_CH(CH_3_)–repeating units	Melting point (°C): 151–166 Thermal decomposition temperature (°C): >240	[139,140]
PI	Characterized by the presence of the imide group in the polymer backbone	Glass transition temperature (°C): 280–400	[141]
PTFE	–CF_2_CF_2_–repeating units	Melting point (°C): 327 Thermal conductivity (W/(m·K)): 0.25 Density (kg/m^3^): 2200	[142]

**Table 8:** Properties of PP, PI, and PTFE polymer membranes.

Materials	Methods	Pore Size (µm)	Flux (L/m^2^·h)	Ref.
PP	TIPS	0.02–0.89	937–7875	[168,169,170]
	Stretching	0.1–3	240–5400	[171,172,173]
	Electrospinning	0.55–0.95	600–5400	[174,175,176]
PI	NIPS	0.06–0.2	0.2–6.4	[177,178]
PTFE	Stretching	0.1–10	1440–142,632	[179,180]
	Spinning	0.01–1	4.2–14.59	[181,182]
	Auxiliary-assistant pore forming	0.01–1	N/A	[183,184]

## Abbreviations

3D	3-dimensional
BR	birefringence
CST	continuous service temperature
DIDP	diisodecyl phthalate
DINCH	di-isononyl-cyclohexane-1,2-dicarb-oxylate
DLP	direct light processing
DOP	dioctyl phthalate
DPE	di-phenylether
EVA	ethylene vinyl acetate
HDPE	high density polyethylene
HMB	hexamethylbenzene
J	flux
LCST	lower critical solution temperature
LDPE	low density polyethylene
L–L	liquid–liquid separation
LLDPE	linear low density polyethylene
LP	liquid paraffin
MSCS	melt-spinning combined with cold-stretching
NIPS	non-solvent induced phase inversion
OPE	oxidized polyethylene
P	permeability
P’	permeance
PDMS	polydimethylsiloxane
PE	polyethylene
PE-b-PEG	polyethylene-block-poly(ethylene glycol)
PI	polyimide
PP	polypropylene
PR	photoresist
PS	polystyrene
PSU	polysulfone
PSµM	phase separation micromolding
PTFE	polytetrafluoroethylene
PTMG	polytetramethyleneglycol
PVDF	poly (vinylidene fluoride)
R	true retention coefficient
RIE	reactive ion etching
R_obs_	observed retention coefficient
SBO	soybean oil
SEPS	polystyrene-block-poly(ethylene-ran-propylene)-block-polystyrene
S–L or L-S	solid–liquid separation
T_1_	initial temperature
TCE	trichloroethylene
TEPTEH	triethylolpropanetris (2-ethylhexanoate)
T_G_	glass transition temperature
TIPS	thermally induced phase separation
T_m_	melting temperature
TPMS	triply periodic minimal surfaces
UCST	upper critical solution temperature
UHMWPE	ultra-high molecular weight polyethylene
V_1_	extrusion speed
V_2_	take-up speed
VIPS	vapor-induced phase separation
α	selectivity factor
β	separation factor
χ	interaction parameter
